# ITAC and Non-ITAC Sinonasal Adenocarcinoma: Classification, Etiopathogenesis, Diagnosis and Therapy Focusing on Interdisciplinarity

**DOI:** 10.3390/medicina61111895

**Published:** 2025-10-22

**Authors:** Miriam Sciacca, Federico Chillari, Stefano Pergolizzi, Valeria Venuti, Giuseppe Iatì, Silvana Parisi, Giuliana Ciappina, Fabio Minutoli, Vincenzo Fiorentino, Guido Fadda, Antonio Bottari, Giacomo Ferrantelli

**Affiliations:** 1 Radiation Oncology Unit, Department of Biomedical, Dental Science and Morphological and Functional Images, University of Messina, Via Consolare Valeria 1, 98124 Messina, Italy; miriamsciacca06@gmail.com (M.S.); giuseppe.iati@unime.it (G.I.); silvana.parisi@unime.it (S.P.); giacomo.ferrantelli@outlook.com (G.F.); 2 Radiation Oncology Unit, “Abele Ajello” Hospital, Via Salemi 175, 91026 Mazara del Vallo, Italy; 3 Radiation Oncology Unit, Villa Santa Teresa, Viale Ing. G. Bagnera 14, 90011 Bagheria, Italy; chillari.federico92@gmail.com (F.C.); vvenuti1991@gmail.com (V.V.); 4 Department of Clinical and Experimental Medicine (DIMED), University of Messina, Via Consolare Valeria 1, 98124 Messina, Italy; 5 Department of Medical Sciences, Section of Experimental Medicine, University of Ferrara, Via Fossato di Mortara 70, 44121 Ferrara, Italy; giuliana.ciappina@unife.it; 6 Nuclear Medicine, Department of Biomedical and Dental Sciences and Morpho-Functional Imaging, University of Messina, Via Consolare Valeria 1, 98124 Messina, Italy; fabio.minutoli@unime.it; 7 Department of Human Pathology of the Adult and Developmental Age “G. Barresi”, University of Messina, Via Consolare Valeria 1, 98124 Messina, Italy; vincenzo.fiorentino@unime.it (V.F.); guido.fadda@unime.it (G.F.); 8 Radiology Unit, Department of Biomedical, Dental Science and Morphological and Functional Images, University of Messina, Via Consolare Valeria 1, 98124 Messina, Italy; bottaria@unime.it; 9 PhD School “Precision Medicine”, Department of Discipline Chirurgiche, Oncologiche e Stomatologiche (Di.Chir.On.S.), University of Palermo, Piazza Marina 61, 90133 Palermo, Italy

**Keywords:** ITAC, non-ITAC, sinonasal adenocarcinoma

## Abstract

Sinonasal adenocarcinomas are rare malignant tumors arising from the epithelial lining of the sinonasal tract. They are classified into intestinal-type (ITAC) and non-intestinal-type adenocarcinomas (non-ITAC), with different histopathological features, aetiologies, and prognostic outcomes. Occupational exposure, particularly to wood and leather dust, is strongly linked to ITAC. Diagnosis requires a combination of clinical evaluation, imaging, histology, and immunohistochemical profiling. Due to the complexity of the sinonasal anatomy and the aggressive behaviour of these tumors, an early and accurate diagnosis is fundamental. Treatment usually involves surgical resection, often followed by radiotherapy, while the role of chemotherapy remains limited. This review outlines the classification, etiopathogenesis diagnosis and management strategies for sinonasal adenocarcinomas, emphasizing the importance of multidisciplinary approaches for optimal outcomes.

## 1. Introduction

Sinonasal cancers (SNCs) represent a rare subset of tumors, constituting less than 1% of all neoplasms and less than 4% of those localized in the head and neck region. Predominantly affecting the nasal cavity and paranasal sinuses, including the maxillary, ethmoid, frontal, and sphenoid sinuses, SNCs often manifest with involvement of the maxillary sinus as the primary site, while the nasal cavities are also commonly affected.

These cancers exhibit histological diversity, arising from various tissue types such as epithelial, mesenchymal, or haematolymphoid origins, resulting in a spectrum of presentations including squamous cell carcinoma, adenocarcinoma, fibrosarcoma, and lymphoma [1,2].

Sinonasal adenocarcinomas (SNAC), the second most prevalent malignancy affecting the sinonasal cavities following squamous cell carcinoma (SCC), account for 10–20% of primary sinonasal malignancies. Sinonasal intestinal-type adenocarcinoma (ITAC) and non-intestinal-type adenocarcinoma (non-ITAC) are both infrequent subtypes of adenocarcinoma [3], although they represent the only two reported entities of adenocarcinomas “in nasal, paranasal and skull base tumours” chapter of the 5th edition of WHO of head and neck tumours [4].

Diagnosis of SNCs frequently occurs at advanced stages, posing challenges in identifying the precise origin, with the frontal and sphenoid sinuses less frequently implicated. Accurate diagnosis of sinonasal malignancies requires the integration of morphological, immunohistochemical, and molecular data, including intestinal markers such as CK20, CDX2, MUC2, and SATB2, as well as respiratory markers such as CK7. These parameters are essential to correctly classify ITAC and non-ITAC and to guide therapeutic management [5,6].

Unlike well-researched and common cancer types, there is limited knowledge about sinonasal ITAC/non-ITACs. Currently, there is no tailored treatment strategy for these tumours, with existing regimens borrowed from other histological types. However, tumours with distinct histological characteristics display considerable biological diversity and exhibit differing behaviours. Surgery is the primary approach for sinonasal non-ITACs, often followed by radiotherapy and chemotherapy. In fact, achieving clear surgical margins with complete resection is hardly challenging due to anatomy, thus leading to adjuvant radiotherapy. It is worthy of note that sensitivity to radiotherapy of these tumors remains uncertain. Novel systemic treatments like targeted therapy and immunotherapy, introduced for other head-and-neck cancers, remain largely unexplored in sinonasal ITAC/non-ITACs. Additionally, the limited research on targeting specific proteins or genes complicates the development of new treatment strategies, as the rarity of these cancers makes patient inclusion in clinical trials challenging. While some rare sinonasal malignancies are treated with tumor-specific approaches, these strategies are often borrowed from other tumour sites, such as immunotherapy for sinonasal mucosal melanoma originally developed for cutaneous melanoma [7]. In fact, immunotherapy has achieved major clinical success in the treatment of melanoma, but in sinonasal carcinomas remains less explored in this regard. Nevertheless, increasing evidence suggests that a subset of sinonasal adenocarcinomas expresses immune checkpoint molecules such as PD-L1 and displays tumor-infiltrating lymphocytes, supporting the rationale for immunotherapeutic intervention. In head and neck squamous cell carcinoma, PD-L1 expression has been correlated with response to anti-PD-1/PD-L1 therapy, a finding that may extend to sinonasal malignancies [8,9,10,11,12].

This review aims to explore recent literature to provide a comprehensive overview of this rare clinical scenario, highlighting the importance of multi- and interdisciplinary approaches as a future direction for integrated and synergistic management.

## 2. Diagnosis

The diagnosis of malignant neoplasms of the nasal cavities and paranasal sinuses is made through an otolaryngological examination (physical examination) supplemented by endoscopic techniques that are necessary to visualize the extent of the tumor in the sinonasal area and confirmed by radiological assessments. The histological type is determined through a biopsy and histological examination [13,14].

### 2.1. Clinical Presentation and Epidemiology

The diagnosis is frequently delayed as these tumors either present no symptoms or exhibit nonspecific symptoms during their initial stages, such as nasal obstruction, headache, hypo/anosmia, epistaxis, confusion, and visual loss [3,15].

Intestinal-type adenocarcinoma (ITAC) stands as the second most common form of sinonasal adenocarcinoma, following adenoid cystic carcinoma (AdCC) in prevalence. Distinguished by growth patterns reminiscent of intestinal carcinomas or adenomas, ITAC often mirrors the histological features of normal intestinal mucosa. Predominantly affecting males across a wide age range, typically between 50 and 64 years old, these tumors are primarily found in the ethmoid sinus (40%), followed by the nasal cavity (25%) and the maxillary antrum (20%). Known for their aggressive nature, ITACs demonstrate a tendency to invade nearby structures such as the orbit, pterygopalatine fossa, infratemporal fossa, and cranial cavity [5,16].

Most cases (88%) are attributed to occupational exposures, with wood dust being the primary risk factor, followed by textile industry products. Interestingly, other established risk factors for sinonasal cancer, such as formaldehyde, nickel/chromium compounds, or asbestos, have not been confirmed as contributors to adenocarcinoma development [2,17,18,19]. Abi-Saab et al. recently found an association between HPV infection and sinonasal carcinomas, particularly adenocarcinomas. In these patients, an improvement in overall survival (OS) and progression-free survival (PFS) was observed, although without statistical significance [20]. Sporadic tumors are typically found in women, and the maxillary antrum is the most common site. Furthermore, Sporadic ITACs have a poor prognosis with short survival [21,22].

Regional lymph-node metastases are uncommon at presentation, occurring in fewer than 10% of cases, yet they represent an adverse prognostic factor. Distant metastases, mainly involving the lung and bone, are rare (<5%) at initial diagnosis but may arise in recurrent or advanced disease [23].

### 2.2. Imaging (CT and MRI)

The principal imaging modalities to study the nose and paranasal sinuses are radiograph, Computed tomography (CT) and Magnetic resonance imaging (MRI). The first method is utilized especially in pediatric patients as a first-line approach in case of upper airway pathology.

The main challenge remains the identification of specific features that can ensure a reliable differential diagnosis between ITAC and non-ITAC. Unfortunately, no distinctive radiological characteristics have been clearly established to date. Consequently, radiological signs that may be considered highly suggestive (though not pathognomonic) can only be inferred indirectly, based on the known biological behavior of these two specific—or even other—entities.

CT and MRI are the primary modalities for the assessment of tumor size, nature, extent, and invasion into adjacent structures such as the anterior and middle cranial fossa, orbit, pterygopalatine fossa, palate, and infratemporal fossa, dural/transdural involvement, perineural spread and intraorbital extension. Imaging can also suggest the malignant transformation of benign pathologies on follow-up imaging, by detecting bone destruction, extension outside the sinonasal cavity, or a change in the pattern of enhancement [24].

CT evaluation includes non-contrast and contrast-enhanced phases, which are best evaluated when they are reformatted to include three planes in both soft-tissue and bone algorithms. On CT, sinonasal adenocarcinomas appear as a soft-tissue mass and occasionally exhibit areas of calcification, which reflect the mucin content. In unilateral olfactory cleft adenocarcinomas, the bulging of the nasal septum across the midline and widening of the olfactory cleft are observed [25].

High-grade adenocarcinomas often show bone destruction. Adenocarcinomas arising from the ethmoid sinus may potentially extend to the skull base and intracranially to the frontal lobes [21]. MRI sequences that are best for evaluating nasal masses include thin-section, fat-suppressed T1-weighted and T2-weighted imaging (T2WI), contrast-enhanced T1WI with fat suppression, and diffusion-weighted imaging. It affords precise soft tissue characterization with high-contrast resolution, which discriminates neoplastic tissue from nasal secretions and inflammatory changes, and defines any possible critical extension. On MRI, the signal intensity of adenocarcinomas varies according to their mucin content, cellularity, and presence of hemorrhagic-necrotic areas. Mucin-producing adenocarcinomas usually show spontaneous hyperintensity on T2-weighted images and gradual enhancement on contrast-enhanced.T1-weighted sequences, whereas adenocarcinomas without mucin production show isointensity to hypointensity on T2-weighted images. However, the imaging features of adenocarcinomas are often indistinguishable from those of other sinonasal cancers [26]. Other imaging characteristics are often indistinguishable from SCC.

### 2.3. PET/CT

Only scant scientific literature about nuclear medicine and sinonasal adenocarcinomas exists. ITAC shows high uptake of 18F-FDG [27]. In a study evaluating FDG uptake in different neoplastic diseases, the mean SUVmax in 16 patients with sinonasal adenocarcinoma was high, namely 9.9. Mean SUVmax was similar in patients with malignant melanoma, adenoid cystic carcinoma and olfactory neuroblastoma [28]. On the other hand, low FDG uptake and high 68Ga-FAPI uptake have been reported in a case of head and neck hidradenocarcinoma [29]. 18F-FDG PET imaging has been suggested to detect recurrence during follow-up [30].

In the study by Önner et al., an emblematic case was described that illustrates the diagnostic complexity of sinonasal adenocarcinomas, emphasizing the complementary role of PET/CT imaging in their evaluation. In this brief, but fundamental report, the authors describe a case of 68Ga-PSMA PET/CT-positive-ITAC with a completion MRI, showing at one time the classic localization and morphology of ITACs while pointing out an unusual metabolic feature which is the avidity for 68Ga-PSMA radionuclide [31].

### 2.4. Histopathology

**Figure 1:**
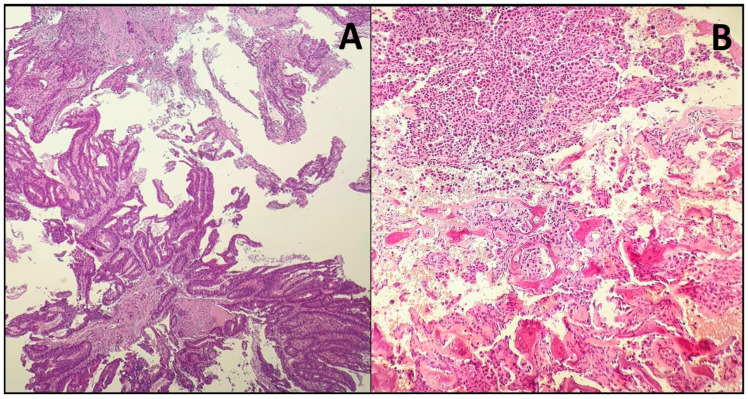
(**A**) Intestinal-type sinonasal adenocarcinoma (ITAC). Low-power view showing complex villous/tubulopapillary architecture with surrounding stroma, simulating intestinal mucosa. The fronds are lined by atypical columnar epithelium with nuclear pseudostratification and intraluminal mucin. Hematoxylin–eosin (H&E). Original magnification ×100. (**B**) Non-intestinal-type sinonasal adenocarcinoma (non-ITAC), low-grade, tubulopapillary pattern. Branching tubulo-papillary structures lined by uniform cuboidal-to-low-columnar cells displaying mild cytologic atypia, inconspicuous nucleoli, and low mitotic activity; background with hemorrhagic debris and scattered inflammatory cells. Overt “dirty” necrosis and overt intestinal differentiation are absent. Hematoxylin–eosin (H&E). Original magnification ×100.

Intestinal-type adenocarcinomas are the more common subtype of non-salivary paranasal cavity adenocarcinoma. They differ from non-ITAC for histopathological characteristics. ITACs are named because of their histologic resemblance to intestinal tumors, which can range from benign polyps to aggressive adenocarcinoma [5]. Most sinonasal non-ITACs are of the low-grade type, whereas high-grade non-ITACs are rare. Although the age of the patients with sinonasal ITAC and non-ITAC may vary widely, patients in the sixth decade of life are most affected. The nasal cavity is the most common site for ITAC and non-ITAC, whereas the paranasal sinuses are less commonly affected [32]. A histopathological imaging comparison between intestinal-type (ITAC) and non-intestinal-type (non-ITAC) adenocarcinomas is presented in Figure 1 [33].

Accordingly to some authors, SNAC encompasses both salivary and non-salivary types based on the cell of origin, with the non-salivary type further divided into intestinal adenocarcinoma (ITAC) and non-intestinal adenocarcinoma (non-ITAC) based on histological characteristics. Further categorization of non-ITAC includes high-grade and low-grade subtypes, while ITAC histologically presents as papillary, colonic, solid, mucinous, or mixed types [16,34].

Mimicking both normal and neoplastic mucosal features of the large and small intestine, ITACs are histologically classified into five main categories: papillary, colonic, solid, mucinous, and mixed types. These subdivisions, further delineated by Kleinsasser and Schroeder, correlate closely with clinical behavior [35,36].

Sinonasal intestinal-type adenocarcinomas (ITACs) histologically resemble adenocarcinomas of the gastrointestinal tract, being composed of a single layer of eosinophilic cuboidal or columnar cells forming glandular structures with a tubular or papillary architecture, similar to colorectal adenocarcinomas [37]. The cells in ITACs often show varying degrees of differentiation, with a high frequency of poorly differentiated characteristics, including high nuclear grade, prominent nucleoli, and increased mitotic activity. Additionally, ITACs may exhibit areas of necrosis, perineural and vascular invasion, which are significantly related to a more aggressive behavior of the neoplasm [38]. Moreover, mucin production is a common feature of such entities, with the presence of intracellular and extracellular mucin that may vary in quantity and distribution within the tumor, and its presence can be confirmed through special stains such as periodic acid-Schiff (PAS) or mucicarmine [37]. ITACs typically express intestinal immunophenotypic markers such as cytokeratin 20, CDX-2, MUC2, SATB2 and villin, and frequently stain positively for cytokeratin 7 (typical of airway epithelia) [39,40,41]. They may also express chromogranin A and other neuroendocrine markers in up to 75% of cases [42]. Only the integration of pathological and clinical information can differentiate ITACs from metastatic colorectal adenocarcinomas, which account for about 6% of secondary sinonasal neoplasms [5].

Non-intestinal-type adenocarcinomas (non-ITACs) are characterized by a heterogeneous histological appearance, including papillary and glandular patterns in low-grade forms and predominantly solid growth patterns in high-grade ones [43]. Examples of non-ITACs are squamous cell carcinomas, esthesioneuroblastomas, salivary-type adenocarcinomas, and sinonasal undifferentiated carcinomas [44]. They typically lack the immunohistochemical expression of the above-mentioned intestinal-specific markers and show a respiratory-type profile with positivity for CK7. Overall, they are considered a diagnostic category of exclusion, with careful attention needed to rule out other possible primary tumors, including metastatic malignancies, although recent studies have identified seromucinous differentiation in many of these tumors [32].

In comparison with non-ITACs, ITACs follow a more aggressive clinical course, showing high-grade features similar to aggressive gastrointestinal adenocarcinomas and presenting at an advanced stage. However, high-grade non-ITACs can also exhibit aggressive behavior, emphasizing the importance of a correct histological classification for the appropriate management of the patients. Table 1 summarises the features comparision between ITAC and non-ITAC.

### 2.5. Tumour Staging

Accurate staging of carcinomas of the nasal cavity and paranasal sinuses is essential for determining prognosis and guiding therapeutic decisions. Staging is performed according to the American Joint Committee on Cancer (AJCC) Cancer Staging Manual, 8th Edition, which provides separate clinical classification (cTNM) for tumors arising in the maxillary sinus versus the nasal cavity/ethmoid sinuses. Both categories have a common pathological classification (pTNM). Clinical N stage describes regional lymph node (cervical nodes) metastasis, which is relatively uncommon at presentation but is a poor prognostic factor. Nodal pathological assessment—with specific regard to extranodal extension—is fundamental in the decision-making process regarding adjuvant radiotherapy (needed/not needed; volumes; doses) and adjuvant systemic therapy. Histological examination of a selective neck dissection specimen will ordinarily include 10 or more lymph nodes. Histological examination of a radical or modified radical neck dissection specimen will ordinarily include 15 or more lymph nodes.

M stage refers to the presence of distant metastases, most commonly to the lungs or bone [4].

A comprehensive clinical, radiological, and histological assessment is essential for accurate staging and the development of personalized therapeutic strategies and should adhere closely to both cTNM and pTNM classifications. Within this context, an interdisciplinary approach—ideally involving ultra-early case discussion—may represent the most effective means of anticipating the therapeutic pathway, minimizing errors, and optimizing outcomes.

For detailed staging criteria, please refer to the AJCC Cancer Staging Manual, 8th Edition (Springer, 2017) [45].

## 3. Therapy

There is a wide range of therapies available for this type of cancer. Surgery is often the first step and can be followed by adjuvant therapies such as radiotherapy, which in some cases may be performed as the initial treatment, and chemotherapy, which is often used in specific conditions.

### 3.1. Surgery

Surgery, when feasible, remains the cornerstone of curative-intent treatment for sinonasal adenocarcinomas and can be complemented with radiotherapy and chemotherapy. The primary objective is to achieve a complete en bloc resection with negative histological margins (R0), as margin status is consistently the most significant predictor of local control and overall survival. However, achieving this is often challenging due to the complex anatomy of the sinonasal cavities and the proximity of critical structures such as the orbit, optic nerves, and anterior skull base. The different surgical approaches can be divided into open surgery, endoscopic surgery, and a combination of both approaches. Furthermore, the neck dissection has a role in some instances. For ITAC, which predominantly arises in the ethmoid sinus and nasal cavity, achieving clear margins can be particularly difficult near the skull base. For high-grade non-ITACs, their aggressive and infiltrative growth pattern presents a similar challenge. Thus, the choice of surgical approach—endoscopic, open, or a combination—is dictated by tumor origin, size, and extension rather than histology alone. Regardless of the technique, reconstruction, particularly of the skull base using vascularized flaps (e.g., pericranial flap), is critical after extensive resections to prevent complications like cerebrospinal fluid (CSF) leaks.

#### 3.1.1. Open (External) Surgery

This approach was the only option for sinonasal cancer before the advent of endoscopic endonasal surgery, which is represented by two main approaches: trans-facial and craniofacial. Nowadays, open or combined cranio-endoscopic approaches are reserved for tumors with extensive invasion into the orbit, dura, brain parenchyma, or skin, where a pure endoscopic approach would compromise the completeness of resection.

- The trans-facial approach can be used for cancer in all sinonasal regions. There are two types of trans-facial approaches: the lateral rhinotomy approach and the sublabial approach.
  ○ Lateral Rhinotomy: The skin incision starts from the medial can, thus, and continues to the nasolabial sulcus and the alar-facial sulcus. This exposes the maxillary sinus, the orbital rim, and the piriform aperture. The zygomatic bone and the maxillary tuberosity can also be exposed through this incision. Depending on the involvement of the infraorbital nerve, it can be preserved or sacrificed.
  ○ Sublabial Approach: The incision is made on the mucosa of the upper vestibule, down to the bone. This approach provides access to the midface skeleton without a skin incision but offers less exposure. There are two types of sublabial approaches: Rouge Denker and degloving.
    ▪ Rouge Denker Approach: The incision is made in the upper vestibular mucosa, exposing the anterior part of the maxilla.
    ▪ Degloving Approach: This involves a bilateral incision from one maxillary tuberosity to the other, providing greater exposure.
- Craniofacial resection is reserved for tumors extending to the anterior skull base, allowing resection of both the lower intrasinusal part of the tumor and any intracranial extension, including orbital invasion. A coronal incision is performed, followed by a bifrontal craniotomy. The frontal lobes are reclined to expose the intracranial tumor. If the cancer has invaded the dura, dissection can be performed intradurally or extradurally. The use of these approaches has decreased in favour of endoscopic surgery [46,47,48].

#### 3.1.2. Endoscopic Surgery

Initially used for benign sinonasal tumors, endoscopic surgery has been extended to malignant ones, representing a valid alternative surgical technique. This approach uses a multi-layer centripetal resection technique based on the tumor insertion area.

The steps are as follows:

- Tumor debulking;
- Identification of the tumor’s adhesion site;
- Tumor removal;
- Expansion of an additional safety plane due to tumor invasion;
- Multiple biopsies for final histological analysis;
- Reconstruction, if necessary.

This approach is performed under general anaesthesia, with the nasal cavity packed with a vasoconstrictive agent beforehand. A 4 mm diameter endoscope with 0° or 30° is used. To avoid massive bleeding, epinephrine and xylocaine can be used. Once the tumor attachment site is identified, tumor debulking begins. Depending on the tumor’s location and extent, different areas may need to be exposed. The tumor is then removed centripetally, from the peripheral area to the attachment zone, as en bloc resection is rarely possible. An important phase is the biopsy for precise mapping of the resection margins for adjuvant radiotherapy [49,50,51,52,53].

#### 3.1.3. Combined Open and Endoscopic Approaches

Combining these techniques allows for the treatment of lesions in all cranial compartments (intra- and extra-cranial) [54,55].

#### 3.1.4. Neck Dissection

Neck dissection is recommended only if node involvement is confirmed by clinical-radiological evaluation. Prophylactic neck dissection (PND) remains controversial, but when performed, it could decrease the risk of regional recurrence [55,56].

### 3.2. Radiotherapy

Radiotherapy is considered in the adjuvant setting. Its indication remains debated for low-stage disease, particularly when wide disease-free margins are present. The treatment policy is not yet fully established for sinonasal intestinal-type adenocarcinomas (ITAC) and non-intestinal-type adenocarcinomas (non-ITACs).

In the literature, the prescribed dose for ITAC tumors is typically 30 fractions of 2 Gy/day to a total dose of 60 Gy, which may be increased with an additional boost of 6 Gy in the case of positive surgical margins. For non-ITAC tumors, the radiation dose is escalated to 66–70 Gy, as this histological subtype appears to be more aggressive, particularly when R1 (microscopic residual) or R2 (macroscopic residual) resections are confirmed. Local control is dose-dependent, but the proximity of organs at risk can result in significant toxicity [57].

Prophylactic cervical irradiation is also valuable for suspicious lymph nodes seen on MRI or CT scans [58,59,60].

Other modalities, such as hadrontherapy, may be considered due to their ballistic advantage—delivering maximum energy at the target site—potentially offering superior dose distribution. Carbon ion radiotherapy is gaining momentum in the treatment of head and neck tumors and is already validated for head and neck and anterior skull base adenoid cystic carcinomas, though it has not yet been validated for ITACs [61].

Another therapeutic option is brachytherapy (BT). Kadah et al. applied this treatment in 20 patients. A small nasal package was inserted into the posterior nasal compartment and removed after 3–5 min to create a personalized BT applicator. BT was delivered using an Ir-192 high dose-rate afterloading technique for 2–5 sessions, reaching a total reference dose of 10 Gy up to 20 Gy. Additionally, EBRT treatment was performed [62].

The definition of tumor and target volumes for radiotherapy is crucial for effective radiotherapy planning. Threshold values for defining treatment fields are based on the guidelines of the International Commission on Radiation Units and Measurements (ICRU) [63].

F. Guillemin et al. in 2023 [64] defined postoperative target volumes using co-registration of preoperative MRI or PET-TC, when possible, to improve volume delineation.

The Clinical Target Volume High Risk (CTV-HR) is defined on preoperative imaging, postoperative MRI if available, with an expansion of 10–15 mm. The Clinical Target Volume Low Risk (CTV-LR) requires a dose of 54 Gy and includes anatomical structures or compartments contiguous to the primary tumor, including, if necessary, fissures, meatuses, canals and pathways of potential spread such as perivascular and perineural routes. In case of uncertainty regarding these extensions, volume enlargement is recommended to minimize the risk of recurrence. The authors suggest excluding air spaces confined within anatomical boundaries from CTV-LR and applied a geometric expansion of 10–15 mm from the preoperative volume. Further expansion may be warranted based on clinical risk and the anatomical segment involved, assessing the anterior, posterior, superior, inferior, medial, and lateral boundaries.

- For tumors originating in the maxillary sinus, the following areas should be included:
  ○ Anteriorly: ipsilateral nasolacrimal duct, anterior wall of the ipsilateral maxillary sinus, labial gingival sulcus, and the maxillary nerve.
  ○ Posteriorly: posterior wall of the infratemporal and pterygopalatine fissures with the ipsilateral process and the foramen ovale, ipsilateral sphenoid sinus, and the foramen rotundum.
  ○ Superiorly: inferior orbital wall, ipsilateral Gasser ganglion; in case of orbital invasion, also the superior orbital fissure and the optic canal.
  ○ Inferiorly: hard palate and the alveolar border.
  ○ Medially: ipsilateral nasal cavity, including the nasal septum.
  ○ Laterally: perijugal fat and the infratemporal fossa.
- For tumors originating in the nasal cavities, these include the following:
  ○ Anteriorly: nasal vestibule, cheek, follow the margin of the nasal bones and the anterior portion of the maxillary nerve.
  ○ Posteriorly: nasopharynx, including the clivus; in large tumors, also the sphenoid sinus and the pterygoid processes.
  ○ Superiorly: ethmoid, pterygopalatine fossa, sphenopalatine foramen, foramen rotundum, inferior orbital wall, and the maxillary sinus.
  ○ Inferiorly: same expansions as for maxillary sinus tumors.
  ○ Laterally: nasolacrimal ducts, ipsilateral maxillary sinus, middle meatus, and pterygoid processes.

### 3.3. Systemic Therapy

The use of systemic therapy in sinonasal adenocarcinomas should be analysed within the context of a complex disease, characterized by different histological subtypes with varying prognoses and various therapeutic options. Furthermore, it is most effectively administered within a multimodal treatment strategy. In this context, the optimal sequencing of various modalities may be crucial in achieving the best possible outcome. Neoadjuvant chemotherapy is aimed at improving distant metastasis control and potentially enhancing local control, while concomitant therapy aims to increase locoregional control. Some studies have evaluated the use of induction chemotherapy in patients with locally advanced sinonasal non-ITAC. Licitra et al. conducted a phase II study to evaluate the efficacy of primary systemic chemotherapy using cisplatin, fluorouracil, and leucovorin (PLF combination) followed by surgery and radiotherapy in patients with paranasal cancer. They observed a pathological complete remission rate of 16% (8 out of 49 patients) [65]. In another study focusing on sinonasal ITAC, Bossi et al. compared the 5-year overall survival (5-year OS) and disease-free survival (5-year DFS) of two treatment groups. One group (Group A) consisted of 30 patients treated with surgery followed by radiotherapy, while the other group (Group B) comprised 44 patients treated with induction chemotherapy using the PLF combination, followed by surgery and adjuvant radiotherapy. Group B demonstrated a significantly higher 5-year OS rate (70% vs. 42%, *p* = 0.041) and 5-year DFS rate (66% vs. 40%) compared to Group A [66]. In addition, it has been observed how the functional status of p53 affects both prognosis and response to neoadjuvant chemotherapy, especially in ITACs. In fact, non-functional p53 appears to correlate with a poorer prognosis and a reduced response to chemotherapy in terms of OS and DFS [66,67,68].

In recurrent and metastatic sinonasal adenocarcinomas, a similar systemic therapy regimen is employed. All treatment regimen in a palliative setting is based on therapeutic schemes used in squamous cell carcinoma of the head and neck and adenocarcinoma of the gastrointestinal tract [69,70].

In sinonasal ITACs, another potential use of chemotherapy in an adjuvant setting is represented by topic application of 5-fluorouracil once/twice weekly for 4–6 weeks following debulking surgery [71,72,73].

Several studies have been conducted to search for target mutations to design specific therapies aimed at improving clinical outcomes, especially in sinonasal ITACs. These studies identified a mutational profile characterized by a low incidence of mutations in the HER2, K-RAS, BRAF, and EGFR genes, a high rate of EGFR copy number amplification and MET and H-RAS mutation, suggesting the potential benefit of anti-EGFR drugs, MET inhibitors, and RAS or MAPK/ERK pathway inhibitors. Another interesting mutation has been observed in the IDH 1 and 2 genes [7,74,75,76].

Riobello et al. evaluated PD-L1 expression as a marker for immunotherapy in 179 cases of sinonasal SCC and ITAC, also examining its potential role as a prognostic factor. They found that PD-L1 was expressed in over 5% of tumor cells in 34% of sinonasal SCC cases and 17% of ITAC cases. PD-L1 expression in more than 50% of tumor cells was more common in sinonasal SCC (26%) compared to ITAC (3%). However, PD-L1 expression did not correlate with clinicopathological parameters and was not an independent risk factor for survival. Although PD-L1 positivity does not appear to have prognostic value, a subset of patients with sinonasal SCC and ITAC might benefit from immune checkpoint inhibitor therapy, which has recently been approved for head and neck cancer [8,77]. An important consideration, however, is that PD-L1 expression can be dynamic. A systematic review on head and neck squamous cell carcinoma by Girolami et al. has shown that conventional radio-chemotherapy can induce heterogeneous changes in PD-L1 levels [11]. This potential for modulation by prior therapies adds a layer of complexity when interpreting PD-L1 status as a predictive biomarker in sinonasal malignancies.

### 3.4. Post-Treatment Surveillance

Given the high propensity for local recurrence, long-term post-treatment surveillance is mandatory for patients with sinonasal adenocarcinoma. The goal of follow-up is the early detection of recurrent or metastatic disease. A standardized protocol typically includes:

- Clinical Examination with Nasal Endoscopy: Performed every 3–4 months for the first two years, every 6 months for years three to five, and annually thereafter. This allows for direct visualization of the surgical cavity.
- Cross-Sectional Imaging: Contrast-enhanced MRI or CT scans of the primary site and neck are recommended every 6–12 months for the first five years to detect deep or submucosal recurrences not visible on endoscopy.
- Systemic Imaging: Chest imaging (e.g., CT scan) should be considered annually to screen for distant metastases, particularly in patients with high-grade or advanced-stage tumors.

This surveillance schedule should be maintained for life, as late recurrences can occur more than five years after initial treatment.

## 4. Discussion

Sinonasal adenocarcinomas, particularly the intestinal-type (ITAC) and non-intestinal-type (non-ITAC), represent a rare but challenging group of malignancies due to their anatomical complexity, histological heterogeneity, and limited therapeutic standardization. This review underscores the importance of a multidisciplinary diagnostic and therapeutic approach, highlighting both progress and critical gaps in current knowledge.

From an epidemiological standpoint, the strong association between ITAC and occupational exposures, especially to wood dust, is well established [2,17]. The predominance of ITAC in male woodworkers and its frequent localization in the ethmoid sinus reinforce the need for occupational screening programs in at-risk populations [18,25]. Despite these correlations, sporadic cases—particularly among women—remain poorly understood and carry a worse prognosis [22]. Histopathologically, the differentiation between ITAC and non-ITAC is crucial for treatment decisions and prognosis. ITACs share morphological and immunophenotypic similarities with gastrointestinal adenocarcinomas, frequently expressing CK20, CDX2, and MUC2 [37,39,40,41]. However, the diagnosis may be challenging due to overlapping features with metastatic tumors and other sinonasal malignancies [78]. Non-ITACs, on the other hand, are a more heterogeneous group with lower immunophenotypic specificity and variable aggressiveness [32,43]. The lack of uniform classification criteria for non-ITAC subtypes remains a critical limitation in the field [36]. Therapeutically, surgery remains the mainstay of treatment. While open and combined surgical approaches offer radical resection for advanced lesions, endoscopic surgery has shown promising outcomes with lower morbidity and comparable oncologic control in selected cases [47,49,53]. Nevertheless, achieving negative margins remains difficult due to anatomical constraints, often necessitating adjuvant radiotherapy [55,57]. The role of radiotherapy is better defined for ITAC, especially in the postoperative setting, where doses of 60–66 Gy improve local control. The precise delineation of target volumes using preoperative imaging, as described by Guillemin et al., is crucial in optimizing dose distribution and reducing toxicity [57]. Particle therapy (e.g., carbon ions) is a promising avenue given its superior conformality, though evidence in ITAC/non-ITAC is still emerging [61]. Systemic therapy is not yet standardized. Induction chemotherapy with cisplatin, fluorouracil, and leucovorin (PLF) has shown potential benefits in locally advanced disease, especially in improving resectability and long-term survival [64,65]. The predictive role of TP53 status in ITAC, associated with chemotherapy sensitivity, highlights the growing importance of molecular profiling [66,67]. Emerging data suggest that sinonasal adenocarcinomas harbor molecular alterations amenable to targeted therapy, such as MET and EGFR amplification, and IDH1/2 mutations [74,76]. However, the rarity of these tumors has limited the development of robust clinical trials. Similarly, immunotherapy, though promising in head and neck squamous cell carcinomas, has shown variable PD-L1 expression in ITAC/non-ITAC, with unclear predictive significance [79,80].

This underscores a broader issue: the paucity of prospective data and reliance on retrospective series or extrapolations from other head and neck cancers. Multicenter registries and international collaborations are essential to overcome low incidence barriers and provide stronger evidence for therapeutic decisions [23].

The management of these rare and complex tumors epitomizes the necessity of a cohesive, interdisciplinary approach, which is best delivered through a multidisciplinary tumor board (MDT). This collaborative forum is essential for formulating an optimal, personalized treatment plan. The radiologist’s role is to precisely delineate tumor extent and invasion of critical structures, which is fundamental for both surgical planning and radiotherapy contouring. The pathologist provides the definitive diagnosis, including histological subtype (ITAC vs. non-ITAC) and grade, which directly influences prognosis and the intensity of adjuvant therapy. The head and neck surgeon assesses resectability and performs the primary resection, while the radiation and medical oncologists contribute to decisions regarding the sequencing of radiotherapy and systemic therapy, respectively. This integrated decision-making process ensures that all diagnostic and therapeutic facets are considered, from achieving clear surgical margins to minimizing long-term treatment-related toxicity, ultimately leading to improved patient outcomes.

Finally, while advancements in endoscopic techniques, imaging, and molecular diagnostics are reshaping the diagnostic and therapeutic paradigm, the integration of multidisciplinary expertise remains key to optimizing outcomes. Collaboration among pathologists, surgeons, radiation and medical oncologists is not just beneficial but necessary in managing these rare and complex entities.

## 5. Conclusions

Sinonasal ITAC/non-ITAC, though rare, present significant diagnostic and therapeutic challenges due to their anatomical location and variable histological presentations. A thorough understanding of their classification and etiological factors, particularly occupational exposures, is essential for timely diagnosis and effective treatment. Surgery remains the cornerstone of management, with adjuvant radiotherapy often improving local control. Future advancements in molecular diagnostics and targeted therapies may offer new hope for improved prognostication and treatment personalization. Continued research and collaborative clinical efforts are needed to enhance outcomes for patients with these uncommon malignancies.

## Figures and Tables

**Table 1:** Comparative features of intestinal-type (ITAC) and non-intestinal-type (non-ITAC) sinonasal adenocarcinomas.

	Intestinal-Type Adenocarcinoma (ITAC)	Non-Intestinal-Type Adenocarcinoma (Non-ITAC)
Epidemiology & Etiology	Strongly associated with occupational exposure to wood and leather dust; predominantly affects older males.	More heterogeneous; May be sporadic or linked to general carcinogens. Divided into Low-Grade and High-Grade.
Common Anatomic Site	Ethmoid sinus (most common), followed by the nasal cavity.	Nasal cavity, maxillary sinus.
Histopathology	Resembles colorectal adenocarcinoma; forms glandular, papillary, colonic, solid, or mucinous patterns.	Low-Grade: Papillary or glandular patterns with minimal atypia. <br> High-Grade: Solid growth patterns with significant nuclear pleomorphism and necrosis.
Key Immunophenotype	CK20+, CDX2+, MUC2+. Often CK7-.	CK7+, CK20-, CDX2-. (Respiratory-type profile).
Prognosis	Generally considered aggressive with a high risk of local recurrence.	Low-Grade: Relatively indolent course. High-Grade: Aggressive clinical behavior, similar to ITAC.
Primary Treatment	Surgical resection followed by adjuvant radiotherapy.	Low-Grade: Surgical resection; adjuvant radiotherapy may be omitted if margins are widely negative. High-Grade: Surgical resection and adjuvant radiotherapy.
Adjuvant Radiotherapy Dose	Typically 60 Gy, with a possible boost to 66 Gy for positive margins.	Typically escalated to 66–70 Gy due to higher perceived radioresistance, especially in high-grade subtypes.

## Data Availability

No new data were created or analyzed in this study. Data sharing is not applicable to this article.

## Abbreviations

The following abbreviations are used in this manuscript:

ITAC	Intestinal-type adenocarcinoma
SNCs	Sinonasal cancers
SNAC	Sinonasal adenocarcinoma
SCC	Squamous cell carcinoma
CT	Computed tomography
MRI	Magnetic resonance imaging
18F-FDG	Fluorine-18-fluorodeoxyglucose
68Ga-FAPI	Gallium-68 Fibroblast Activation Protein Inhibitor
PET	Positron emission tomography
AdCC	Adenoid cystic carcinoma
HPV	Human papilloma virus
PFS	Progression free survival
PND	Prophylactic neck dissection
BT	Brachytherapy
ICRU	International Commission on Radiation Units and Measurements
CTV-HR	Clinical target volume-high risk
CTV-LR	Clinical target volume-low risk
PLF	Platinum-based chemotherapy, leucovorin, fluorouracil
OS	Overall survival
DFS	Disease free survival
